# Transmission of parasites to people via food: how can we determine their impact and why do we need to know?

**DOI:** 10.1016/j.fawpar.2025.e00258

**Published:** 2025-03-13

**Authors:** Lucy J. Robertson

**Affiliations:** Parasitology, Department of Paraclincal Sciences, Faculty of Veterinary Medicine, Norwegian University of Life Sciences, Ås, Norway

**Keywords:** Amoebic liver abscess, *Cyclospora cayetanensis*, Disability-adjusted life year, Disease burden, *Trypanosoma cruzi*

## Abstract

Foodborne parasites exert a considerable burden on human health, and this can be estimated using the disability-adjusted life year metric, enabling comparisons across regions, age groups, and among parasites and other foodborne hazards. Previous estimates of the burden exerted by different foodborne hazards were published about a decade ago (2015) and an update is planned. These estimates are important, not only for comparative purposes, but also to provide an evidence-based framework for resource allocation for different interventions, including prioritisation of risk management efforts, determining where food safety policy infrastructure should be focused, financing research and innovation, and for targeting supplier and consumer education.

Here the potential for improving the previous estimates is discussed, not only by using more recent data, but also by inclusion of foodborne parasitic diseases and disease states that were omitted in the previous estimates. In particular, for example, the inclusion of foodborne infection with *Cyclospora cayetensis* and with *Trypanosoma cruzi* are suggested, along with consideration of disease states such as amoebic liver abscess in foodborne amoebiasis. In addition, knowledge gaps, potential interventions, and how intervention effects can be assessed are discussed, using specific examples.

## Highlights

• The health burden associated with foodborne diseases can be estimated in DALYs.
• These provide an evidence-based framework for prioritizing resource allocation.
• Estimates made for 2010, including for foodborne parasites, are being updated.
• Further parasites and disease-states can be assessed for inclusion in new estimates.
• Identifying knowledge gaps and potential interventions are also important outcomes.

## 1 Introduction

Some parasites are only transmitted to people via the foodborne route of infection; these are exclusively parasites whose transmission stage is in some manner encapsulated in the flesh of the previous host. If the transmission stage survives any preparation methods prior to consumption (cooking, freezing, salting, pickling etc.), then the human host may be infected when the flesh of the previous host is eaten, along with the parasite transmission stage. Examples of these parasites are *Trichinella* spp. (transmitted to people in the flesh of an animal such as a pig) and Opisthorchiidae (transmitted to people in the flesh of freshwater fish such as carp).

Some parasites, typically those whose transmission stage is shed in the faeces of the previous host, may be transmitted by food, predominantly fresh produce that is eaten raw such as salad vegetables or fruits. However, they may also be transmitted by other routes, such as hand-to-mouth, via ingestion of contaminated water, or ingestion of contaminated soil or sand. Examples of such parasites are *Cryptosporidium* spp., *Echinococcus* spp., and *Ascaris lumbricoides.*

And some parasites may have a variety of transmission routes, of which oral transmission is only one, and food may not even be widely recognised as a vehicle by which people may become infected. A good example of such a parasite is *Trypanosoma cruzi*.

It should be noted that many of these foodborne parasites are zoonotic or potentially zoonotic, not only involving complex transmission routes and epidemiology, but also, potentially, representing an animal health/production burden, which may, in turn, impact on the household and the wider community.

Thus, determining the extent to which particular parasites are transmitted to people by food, rather than by another route, may be a relatively easy task (particularly for those parasites that are always foodborne). However, for those parasites that can be transmitted by any one of a variety of routes, determining the proportion of infections that are foodborne may be an almost impossible task, varying according to a range of factors such as culinary habits, geographical or climate factors in a particular location, extent and effectiveness of infrastructure such as sanitation, as well as the data collection in the region under investigation.

Attempts to estimate how much foodborne transmission of parasites occurs is not just an academic exercise. Similarly, estimating the associated impact of these infections, in terms of the health and prosperity of populations, is of more than simple intellectual interest. Data from such approximations can be used to inform where resources can be best directed to improve the health of different populations, and also can be used as a basis towards meeting some of the United Nations (UN) Sustainable Development Goals (SDG). These were developed at the UN Sustainable Development Summit in September 2015 and are described as an urgent call for action by all countries in a global partnership that can be used to inspire changes that will combat poverty and other deprivations. Of significance to estimating foodborne disease burden is specifically SDG-3 (good health and well-being), but others are also relevant, including SDG-2 (zero hunger), SDG-6 (clean water and sanitation), SDG-10 (reduced inequalities), SDG-14 (life below water), SDG-15 (life on land), and SDG-17 (partnerships for the goals) (Goh et al., 2024).

Thus, in brief, by making evidence-based estimates of the health burden (incidence, deaths, morbidity (disability-adjusted life years, DALYs, composed of the sum of years lived with disability, YLD, and years of life lost, YLL)), of a foodborne disease, not only due to parasites, but of any specific aetiology, gives impetus to various control measures. These include, but are not limited to: evidence-based risk management and resource allocation; estimates of the economic burden due to these diseases, and thus the potential for cost-benefit analyses regarding interventions; improvements in food safety policies and also in related infrastructure, such as sanitation and water supply; greater and more-targeted consumer education; the possibility to address relative risks and fill data gaps; innovation in sustainable improvement in food production via both terrestrial agriculture and aquaculture; enablement of multi-sectoral/cross-sectoral collaboration, with relevant research and innovation.

For parasites, in particular, perhaps more than for any other aetiology of foodborne disease, determining the extent of transmission, and by which pathway is difficult, especially, as previously noted, many foodborne parasites are zoonotic. There are various reasons for this, partly driven by political will and other national and international priorities. However, in general, foodborne illness is usually equated with acute enteric disease, usually of bacterial aetiology, sometimes viral or chemical. Parasitic diseases, however, are sometimes not considered as being the cause of a foodborne illness as many of them do not fit this pattern. Thus, although some potentially foodborne parasites do indeed result in acute enteric disease (e.g., *Cryptosporidium*), others may result in acute, non-enteric illness (e.g., *Trypanosoma cruzi*), and many more can have a more insidious, long-term effect, which, nevertheless, can have a profound impact on human health, including fatalities (e.g., *Echinococcus* spp., *Paragonimus* spp., *Ascaris lumbricoides*).

**Fig. 1:**
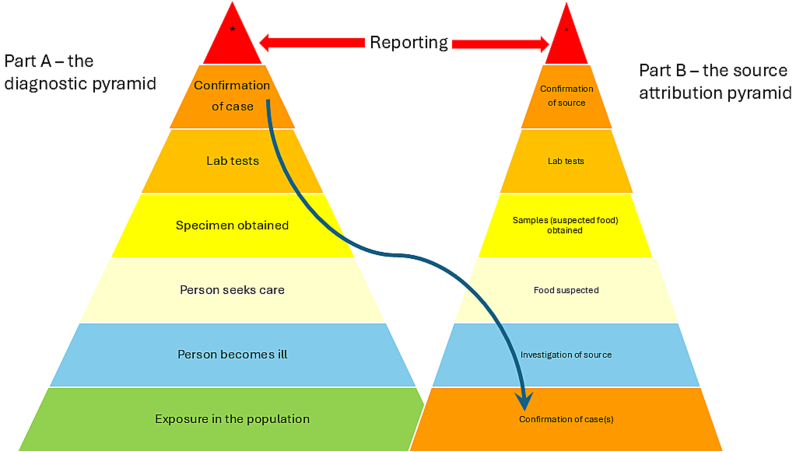
The (steep) diagnostic and (very steep) source attribution pyramids for foodborne parasitic diseases.

The diagnostic pyramid (see Fig. 1, Part A) is therefore often particularly steep for parasitic foodborne disease, and even more so for the source-attribution pyramid (Fig. 1, Part B). The latter is often associated, to some extent, with the relatively prolonged period between ingestion of the food of relevance and the commencement of symptoms, or, even more prolonged should diagnosis be delayed (which is frequently the case). This means that the potential food vehicles are often not available for analysis, having either been consumed or discarded, and relevant foods may not even be recalled by the patient due to the time interval, which may be months or even years, between ingestion and disease.

The main focus of this article is concerned with updating the estimates of the public health burden associated with foodborne parasitic diseases; previous estimates were published approximately a decade ago (Havelaar et al., 2015; Kirk et al., 2015; Torgerson et al., 2015). In particular, in the current article emphasis is placed on where improvements in these estimates can be made rather than simply providing updates based on more recent data. This can be by considering particular parasitic-infection associated health states that were not included in the previous estimates, or even by including specific parasites that were omitted in 2015. Difficulties in making useful estimates and identifying important gaps in our knowledge are also mentioned, along with how such data may be translated into potential interventions, and how any effect from such interventions can be evaluated.

## 2 Previous estimates of the burden of foodborne parasitic disease

In 2010, the World Health Organization (WHO) initiated a process by which the burdens of foodborne diseases were estimated globally; outputs from this process were published in 2015 (Havelaar et al., 2015; Kirk et al., 2015; Torgerson et al., 2015). Almost simultaneously, WHO, together with Food and Agriculture Organization of the United Nations (FAO), initiated a complementary exercise, that only considered foodborne parasites rather than all foodborne hazards, to respond to the request of the Codex Committee on Food Hygiene (CCFH) to review the current knowledge status on parasites in food and their public health and trade impact. The intention was to provide CCFH with advice and guidance regarding the parasite-commodity combinations that should be of particular concern, issues that should be addressed by risk managers, and the available options. The output of this exercise was a prioritisation based on multi-criteria risk-ranking that was published in 2014 (one year before the disease burden estimates). Although this document provided a basis for comparison between the various impacts of different foodborne parasites, burdens of disease associated with particular parasites were not estimated in this endeavour (FAO and WHO, 2014).

Meanwhile, for the foodborne disease burden estimates published in 2015, the data presented were based principally upon a three-step procedure in which first a systematic review of the published literature was conducted to obtain key quantitative information (incidence of infection, mortality, incidence of disease states/sequelae) for selected foodborne diseases both globally and in different world regions. The proportions of these infections that were foodborne were then estimated by hazard-specific source attribution, using either published data or by expert knowledge elicitation (Hald et al., 2016). Thereafter, using disability weights (DALYs) per disease state (largely based on data from elsewhere, particularly those used by the Institute for Health Metrics and Evaluation (IHME) at the University of Washington, USA), the global and regional burdens of 31 foodborne diseases were calculated (Devleesschauwer et al., 2015). The framework for this procedure was described in detail and published separately to the estimated burdens from the various foodborne hazards (Devleesschauwer et al., 2015).

These estimates for 2010, included the burden caused by different pathogens (Kirk et al., 2015; Torgerson et al., 2015): two viruses that may cause foodborne disease, namely hepatitis A virus and norovirus (an invasive enteric disease and a diarrhoeal disease, respectively); 16 bacteria that may cause foodborne disease (either diarrhoeal disease, invasive enteric disease, or intoxications), namely *Campylobacter* spp., enterotoxigenic *E. coli* (ETEC), enteropathogenic *E. coli* (EPEC), nontyphoidal *Salmonella enterica*, *Shigella* spp., *Vibrio cholerae*, Shiga-toxin producing *E. coli*, *Bacillus cereus*, *Clostridium perfringens*, *Clostridium botulinum*, *Staphylococcus aureus*, invasive non-typhoidal *Salmonella enterica*, *Mycobacterium bovis*, *Brucella* spp., *Listeria* spp., *Salmonella enterica* serotype typhi, and *Salmonella enterica* serotype paratyphi A, B, or C; and 14 parasites that may cause foodborne disease (either diarrhoeal disease or invasive infectious disease), namely *Cryptosporidium* spp., *Entamoeba histolytica*, *Giardia duodenalis*, *Toxoplasma gondii*, *Echinococcus granulosus*, *Echinococcus multilocularis, Taenia solium, Ascaris lumbricoides*, *Trichinella* spp., *Clonorchis sinensis, Fasciola* spp., *Opisthorchis* spp., and *Paragonimus* spp.

Major findings from this undertaking were that at that time (2010), approximately 600 million people become sick and 420,000 people die from eating unsafe food, resulting in the loss of 33 million healthy life years (WHO, 2015). Children under 5-years of age were considered to be the population segment most severely impacted, with 125,000 dying from foodborne diseases annually (WHO, 2015). In addition, based partially on these data, a subsequent report from the World Bank (Jaffee et al., 2019), indicated that unsafe food costs over US$110 billion annually in low- and middle-income countries (LMIC), with the costs particularly due to productivity losses and medical expenses. In addition, it was indicated that much of this burden of unsafe food on both health and the economy could be circumvented or reduced through farm-to-fork preventive measures, investments, and modifications in behaviour (Jaffee et al., 2019).

While the median total foodborne disease burden was estimated to be over 30 million DALYs annually, among the foodborne parasitic diseases included in this estimate, the greatest burden was due to cysticercosis (larval infection with *Taenia solium*); as taeniasis is transmitted solely by the foodborne route, all cases of cysticercosis were also classified as foodborne, being consequent to taeniasis (Torgerson et al., 2015). In addition, the combination of the foodborne trematode infections contributed an approximate median of 2.0 million DALYs, and foodborne toxoplasmosis an estimated median of 825,000 DALYs annually (Torgerson et al., 2015). Although foodborne parasitic diseases can be fatal (years of life lost, YLL), the morbidity associated with these infections (years lived with disability, YLD) provided the greatest contribution to the DALY burden (Torgerson et al., 2015).

Cysticercosis had the fourth highest estimated burden of all the hazards considered, with an estimated median annual burden of 2.78 million DALYs; the three foodborne diseases calculated to be associated with even greater burdens were non-typhoidal *S. enterica* infections (both diarrheal and invasive disease) that resulted in 4.07 million DALYs, invasive *Salmonella enterica* Typhi (3.72 million DALYs), and enteropathogenic *E. coli* (2.94 million DALYs) (Kirk et al., 2015).

## 3 Updating the estimates

Although the foodborne DALY estimates calculated by WHO and published in 2015, provided a snapshot of the relative burdens of different foodborne pathogens, there were various weakness inherent in the estimates. These include that data are simply more easily available for some pathogens than others, and therefore for some pathogens the incidence and burden are either grossly under-estimated, or the data are simply not there. This is particularly so for regions or countries with a range of challenges that may easily be prioritised above collating data regarding the incidence of different diseases or the prevalence of pathogens in the population. As parasites have long been considered to be particularly neglected pathogens (Robertson, 2018) and their impacts are often more chronic than acute, it is likely that lack of data is an important concern; this may apply to other hazards also, but for parasites is probably an even greater issue.

As already noted in the previous foodborne disease burden estimates, the burden that was associated with parasites was mostly due to morbidity rather than mortality; although some foodborne parasites may result in a fatal outcome, most have a more insidious impact. Furthermore, many parasites are associated with poverty, and the populations that are most exposed to foodborne parasites are often marginalized, living in the poorest parts of the world, where water supply, sanitation, housing, and transport may be inadequate. In 2020, WHO published a roadmap for neglected tropical diseases (NTD), where among the 20 diseases (or groups of diseases) listed, 12 (60 %) are parasitic, and of these five (42 %) have the potential to be transmitted by food (WHO, 2020). These five diseases or disease groups are: Chagas disease, echinococcosis, foodborne trematodiases, soil-transmitted helminthiases, and taeniasis & cysticercosis.

However, even in highly developed countries, the importance of foodborne parasites is beginning to be recognised, as improved interventions against other pathogens, both in the food-chain and in the healthcare setting, diminish the importance and impact of these other agents. For example, a public health, risk-prioritisation exercise for foodborne pathogens was conducted in 2019 in Norway (one of the wealthiest countries globally) (Norwegian Scientific Committee for Food and Environment (VKM), 2021; Robertson et al., 2024b). This was on request from Norway's Food Safety Authority (NFSA), and included 20 agents of which 12 were bacteria (*Bacillus cereus*, *Campylobacter* spp., *Clostridium botulinum*, *Clostridium perfringens*, enterohaemorrhagic *E. coli* (EHEC), other pathogenic *E. coli, Listeria* spp., *Salmonella* spp., *Shigella* spp., *Staphylococcus aureus*, *Vibrio* spp., and *Yersinia enterocolitica*), five were parasites (members of the Anisakidae, *Cryptosporidium* spp., *Echinococcus multilocularis, Giardia duodenalis*, and *Toxoplasma gondii*), and three were viruses (hepatitis A virus, hepatitis E virus, and norovirus). Thus, there was some overlap in this exercise with the WHO choice of foodborne pathogens for burden estimates from 2010, but not an exact match. This was because the NFSA focussed on those pathogens of relevance to Norway (thus excluding pathogens more associated with other global regions, such as, for example, *Brucella* spp. and *Paragonimus* spp.) and also excluded those foodborne infectious agents that were already addressed by regulated food-control programmes in Norway (such as, for example, *Trichinella*, *Taenia saginata*, and *Mycobacterium bovis*).

Expert knowledge elicitation was used for ranking the 20 foodborne pathogens in Norway, using a scoring system for six criteria concerned with prevalence, morbidity (acute and chronic) and mortality, along with likelihood of the burden of human disease increasing in the future (VKM, 2021; Robertson et al., 2024b). In this wealthy country, where the infrastructure is of high quality and healthcare provision is free, two of the parasites, *T. gondii* and *E. multilocularis*, were ranked very highly (in 1st and 3rd place, respectively). To some extent this may reflect that the other pathogens are more easily diagnosed and treated in the Norwegian healthcare system, as well as being more easily detected in the food chain. Although *Cryptosporidium* is being increasingly recognised as an important pathogen in Norway (e.g., Campbell et al., 2022; Tipu et al., 2024), it ranked about midway (9th place), possibly due to being generally a relatively brief, acute infection in a wealthy country with low AIDS prevalence, with low mortality despite treatment challenges and a relatively high frequency of associated hospitalization. Both *G. duodenalis* and Anisakidae ranked relatively low (15th and 20th place, respectively), again likely due to their limited severity and the ready availability of treatment. Of interest, is not only that two of the parasites scored very highly regarding their public health impact, but also that the parasites included in the assessment were considered more likely to present an increasing foodborne disease burden in the future than the other pathogens; this is not an aspect that is considered in the WHO burden assessment that is based solely on current status and impact.

At the 73rd World Health Assembly in 2020 (World Health Assembly, 2020), it was documented that all 194 WHO Member States requested WHO to monitor regularly, and to report to Member States on, the global burden of foodborne and zoonotic diseases at national, regional and international levels, and, in particular, to prepare, by 2025, a new report on the global burden of foodborne diseases with up-to-date estimates of mortality, as well as incidence, and burden in terms of DALYs. Furthermore, it was requested that progress made in implementing this resolution should be reported to the 75th World Health Assembly. This mandate not only provides the opportunity for bringing the estimates up to date, but also the chance to improve those estimates. This can be, for example, by incorporating data obtained by more sensitive diagnostic techniques that were used less frequently when the previous estimates were made (such as molecular methods for diagnosing *Cryptosporidium* infection, rather than relatively insensitive microscopy), considering the burden associated with disease states or sequelae associated with particular hazards that were not incorporated in the earlier estimates, and estimating burdens for some important foodborne parasites (and other hazards) that were excluded from the previous estimates.

In the following sections, some examples are given of where estimates of foodborne disease burden associated with parasites may be improved by addressing some of these aspects.

### 3.1 Updating the estimates: should a greater disability weighting be used for *Entamoeba histolytica*?

Among the parasites that were included in the 2010 estimates, *E. histolytica* was classified as causing “diarrhoeal disease” and was not included as an “invasive enteric disease”. Although, *E. histolytica* can indeed cause diarrhoea, it also causes various invasive diseases, from amoebic colitis (in the intestine, but which, untreated, can lead to perforation of the colon, fulminant colitis and toxic megacolon; Ximénez et al., 2009) to extra-intestinal invasive disease such as amoebic liver abscess (ALA). According to Cui et al. (2019), approximately 10 % of cases of people infected with this parasite have invasive amoebiasis, and an older publication, in which 20 asymptomatic carriers were followed for over a year, indicated that around 10 % of these subsequently developed invasive forms of disease (Gathiram and Jackson, 1987). In addition, a study from Bangladesh found that among 127 children infected with *E. histolytica*, 10 (8 %) developed invasive disease (Haque et al., 2002).

Given the heavier burden associated with invasive amoebiasis, particularly ALA, inclusion of invasive *E. histolytica* infection in the estimates of foodborne disease burden would mean a significant rise in value. Indeed, a systematic review study from France, inspired by a case series of 15 adult males with hepatic amoebiasis associated with travel abroad (Nasrallah et al., 2022), identified the occurrence of intestinal, hepatic, pulmonary, hepatopulmonary, genital, cerebral, cutaneous, and splenic amoebiasis, although several of the latter were individual case reports. Of particular note, perhaps, were that deaths were infrequently reported. Regional differences were clear, however, with particular hotspots, such as in Thua Thien Hué in central Vietnam where the incidence of ALA has been reported to be over 20 cases per 100,000 inhabitants per year (Blessmann et al., 2002). A longitudinal study over 15 months conducted in this region also indicated that of 43 asymptomatic adult carriers of *E. histolytica*, one (2.3 %) developed ALA during the observational period (Blessmann et al., 2003). Southern and Central America, particularly Brazil, Colombia, Ecuador and Mexico, are also considered to be hotspots both for intestinal amoebiasis and invasive amoebiasis including ALA (Nasrallah et al., 2022), with the incidence of ALA of around 4–10 individuals among 100,000 residents, and this has been suggested to perhaps reflect a particularly virulent strain of *E. histolytica* in this world region (Ximénez et al., 2009).

Although largely associated with LMIC, amoebiasis, potentially with invasive disease, can also occur in wealthier countries, often associated with travel. Of a party of 12 Australians (6 adults and 6 children) who travelled to Timor-Leste, three adults acquired *E. histolytica* infection, two of whom developed ALA (Nourse et al., 2016). The authors propose that a foodborne transmission route is likely as they suggest that the reason only adults became infected may reflect that the children were reluctant to eat the locally prepared vegetables.

It is therefore clear that including a higher disability weighting into a proportion of the cases of foodborne cases of amoebiasis, reflecting those who develop invasive disease, will likely provide a better representation of the burden of disease associated with this parasite.

### 3.2 Updating the estimates: should the burden associated with fishborne Anisakid infections be included?

The burden of foodborne disease due to nematodes in the family Anisakidae (e.g., *Anisakis simplex* and *Pseudoterranova decipiens*) were not included in the previous estimates, as diseases caused by the anisakidae were considered to be uncommon and therefore removed from the priority list (Torgerson et al., 2015).

However, infection with anisakid nematodes has been described as “emerging”, with the reported prevalence increasing globally, particularly in European countries (Cong and Elsheikha, 2021), incorporating not only anisakiasis (anisakidosis) – the disease directly due to the infection with the nematode – but also clinical cases of allergy. In these cases, the allergens (some of which are thermostable) on the parasite surface elicit an allergenic reaction from the consumer, even if the nematodes themselves are dead and thus non-infectious or even removed, although clinical investigations have indicated that live larvae should be present for acute allergic IgE-mediated symptoms to be induced (Daschner and Cuéllar, 2020). Symptoms reported include urticaria, angioedema, or anaphylaxis (gastro-allergic anisakiasis) and also generalized reaction with cutaneous (urticaria-angioedema), and/or respiratory (rhinitis and asthma) symptoms, that often occur in conjunction with abdominal symptoms (nausea, vomiting, or epigastric pain), but may remain mild (Daschner and Cuéllar, 2020).

Although consumption of raw fish has increased worldwide, and thus the occurrence of Anisakid infections may be expected to mirror this, many countries have regulations to mitigate this. For example, it is compulsory in many countries for fresh fish to be used in sushi (for example) to be frozen (−20 °C) for at least 24 h; this was presumably the reason for the low ranking of this parasite in the Norwegian prioritisation task (VKM, 2021; Robertson et al., 2024a); nevertheless, experimental data suggest some anisakids survive freezing, partly dependent on the nature of the fish product (Podolska et al., 2019). However, as previously described, inactivation of the parasite would not necessarily mitigate against clinical cases of allergy associated with this parasite. Indeed, a systematic review suggested that Portugal and Norway were two hot spots for allergenic anisakiasis, with allergy prevalences of over 20 % (Rahmati et al., 2020). However, this was strongly disputed (Daschner et al., 2021), citing also a previous study demonstrating a low seroprevalence of Anisakid sensitization in the Norwegian population (Lin et al., 2014). Thus, although data suggest that this parasite may be increasing in fish and sea mammals (the natural definitive hosts for these parasites), the extent to which this spills over to human infections or allergenic reactions is not clear. Data from a sero-survey in Madrid, comparing levels of anti-*Anisakis* antibodies in 500 blood donors from 2021 to 2023 with equivalent levels from 110 blood donors from 2001 to 2002 (Blanco-Costales et al., 2024), do indeed suggest that exposure is not increasing, with a decrease of over 80 % in the prevalence of anti-*Anisakis* IgE reported.

### 3.3 Updating the estimates: should the burden associated with foodborne cyclosporiasis be included?

Infection with the protozoan parasite *Cyclospora cayetanensis* was excluded from the previous estimates of the foodborne disease burden in 2010 because the frequency of citations regarding this parasite were considered to have remained constant between 1990 and 2008, although were increasing for other intestinal protozoa (*Cryptosporidium, Entamoeba,* and *Giardia*) over that period (Torgerson et al., 2015). Furthermore, one article (Hall et al., 2012) had indicated to these authors (who were involved in selecting the appropriate parasites for the 2010 estimates) that case-numbers of cyclosporiasis over the medium- to long-term were quite low, with a median annual incidence of 0.03 cases per 100,000. It should be noted, however, that this number is based on cases in USA where the vast majority of cases are associated with imported food, rather than in those countries of Latin America where the parasite circulates endemically among the population, and thus this may not have been the most useful article for reaching such a decision. It is also worth noting that although Torgerson et al. (2015) decided that inclusion of the intestinal protozoa *Cryptosporidium, Giardia*, and *Entamoeba* was more relevant than inclusion of the intestinal protozoan *Cyclospora*, the latter may actually be more likely to be foodborne than the other three due to the parasite's requirement to sporulate in the environment at temperatures of between 22 °C to 32 °C for a period of days or weeks after being shed in faeces before it is infective. This means that *Cyclospora* cannot be directly transmitted by hand-to-mouth contact, as can the other three intestinal protozoa, and is therefore more likely spread by an infection vehicle (such as water or food).

Furthermore, for the 5-year surveillance period (2011–2015) in USA, the Centers for Disease Control and Prevention (CDC) reported that the median annual number of reported cases was 398 (mean of 441) ranging from 130 in 2012 to 798 in 2013 (Casillas et al., 2019). However, looking at data from the most recent 5-year period (2019–2023), the median annual number of reported cases was 1241 (mean of 1614) ranging from 1020 in 2021 to 2408 in 2019 (Almeria et al., 2023, based on CDC data until 2023; CDC, 2024). Thus, during the time between the 5-year period from 2011 to 2015 until the 5-year period ending 2023, the median number of cases has approximately tripled. This apparent increase in seasonal outbreaks of cyclosporiasis in USA is relevant, and seasonality appears to be important in the epidemiology of this parasitic disease, although varies by region, probably due to human activities, environmental contamination, and the optimal sporulation conditions. In North America, the majority of cases occur between March and September, peaking around July (Almeria et al., 2023).

However, probably of greater importance than these outbreak events in North America is the impact of these intestinal protozoa in the regions where they are endemic. A recent systematic review of global prevalence of *Cyclospora* infection (Chen et al., 2024) indicates that, despite the large outbreaks in North America, the global prevalence of infection generally increases regionally with decreasing economic status, rising from 0.4 % in high-income countries, to 2.9 % in upper-middle-income countries, to 4.8 % in lower-middle-income countries, to 7.6 % in low-income countries (Chen et al., 2024). Thus, although the health burden per case is unlikely to be substantial, the relatively high occurrence in poorer countries will contribute to the impact. In addition, although not part of the burden calculation used by WHO, the economic impact of *Cyclospora* outbreaks associated with fresh produce exported from poorer countries to wealthier countries, can be substantial and should not be overlooked. An example of this devastating economic impact of foodborne *Cyclospora* infection on exporting countries has been described for the berry industry in Central America (Tefera et al., 2018), when contaminated fresh produce imported to North America was found to be the source of cyclosporiasis outbreaks.

### 3.4 Updating the estimates: should the burden associated with foodborne Chagas Disease be included?

Although foodborne Chagas Disease was considered for inclusion in the previous foodborne disease burden estimates, a lack of resources for commissioning a systematic review for the agent, *Trypanosoma cruzi*, meant that it was omitted (Torgerson et al., 2015). It is possible that the potential significance of foodborne Chagas Disease, although largely regional, was underestimated in reaching the decision not to use resources for this infection; the authors later concluded that other parasites, such as *Giardia* and *Trichinella*, for which resources were used, may be associated with a smaller foodborne burden than foodborne Chagas Disease (Torgerson et al., 2015). Indeed, subsequent papers that have made crude estimates of the disease burden associated with foodborne Chagas Disease (using, as a basis data from IHME; Murray et al., 2012 and Gómez-Ochoa et al., 2022), have suggested a relatively high burden of DALYs (Robertson et al., 2016; Robertson et al., 2024a). In the latter of these (Robertson et al., 2024a), it is noted that the more recent (and lower) crude estimate of foodborne burden of Chagas Disease exceeds that for 11 hazards (7 bacteria and 4 parasites) that were considered of sufficient relevance for resources to be made available for inclusion in the estimates made for 2010 (Kirk et al., 2015; Torgerson et al., 2015).

However, the calculations required for estimating the burden of disease associated with foodborne *T. cruzi* infection are more complicated than for most, if not all, of the other hazards. For the majority of hazards that can be transmitted by more than one route or vehicle (for example, for *Cryptosporidium* the transmission routes to people include person-to-person, animal-to-person (zoonotic transmission), or via ingestion of contaminated water or food) the disability weighting from the disease is the same, regardless of transmission route. Thus, it is relatively simple to determine the DALYs associated with foodborne transmission as a proportion of the total disease burden associated with infection by any transmission route. However, for Chagas Disease there is an increasing body of evidence that indicates that foodborne (or oral) transmission of the infection results in a heavier disability weighting than vectorborne transmission, which is considered the main infection route. As summarised in a review chapter (Alarcón de Noya et al., 2024), unlike in vectorborne Chagas Disease, foodborne infection often results in acute disease, higher symptomatic rates, more serious infections, and greater cardiac involvement in more cases; the outcome may be fatal during this acute phase that manifests relatively rarely when the infection route is by vectorborne transmission. For example, among 230 patients from 10 different foodborne outbreaks of Chagas Disease in Venezuela, fewer than 13 % were largely asymptomatic, around 36 % were moderately ill, and over 50 % were severely ill with 10 deaths (Alarcón de Noya et al., 2015). An investigation of 85 medical records of confirmed cases of acute Chagas Disease associated with oral transmission from the Casanare region of Colombia between 2012 and 2020 reported a fatality rate of over 9 % (8 deaths), with mortality reaching as high as 50 % in some of the foodborne outbreaks (Rincón-Acevedo et al., 2021). Another review of cases of foodborne Chagas Disease (Filigheddu et al., 2017) provides further similar indications of the higher disease burden associated with oral transmission. This article again comments that foodborne Chagas Disease is characterized by more severe acute-phase manifestations than associated with vectorborne transmission, including prolonged fever, acute myocarditis with heart failure, and, occasionally, meningoencephalitis, with mortality rates of over 30 % (Filigheddu et al., 2017). Among the examples cited is one outbreak from Santa Caterina, Brazil in 2005 (Steindel et al., 2008) in which sugarcane juice had been contaminated during preparation, resulting in 24 or 25 cases, including an Italian tourist, and 3 deaths (fatality rate of around 12 %). Thus, both components of the DALY, YLL and YLD, may be larger in foodborne Chagas Disease than in vectorborne disease, thereby complicating the model for estimating burden.

## 4 Possible interventions and how to measure their impact

One main reason for estimating the burden of foodborne disease caused by specific hazards is in order to use these data as an evidence-based tool to prioritize hazard-directed interventions that may act towards reducing the health-burden exerted by these diseases. Some interventions are very generalist and are likely to improve food safety for several hazards. These include measures such as water, sanitation and hygiene (WASH) interventions, food hygiene education (hand washing, keeping food covered), improving diagnostics (multiplex, point-of-care approaches), and targeted and appropriate treatment (for example, not using antibiotics for treating cryptosporidiosis). For those foodborne diseases that cause diarrhoea, and probably many others, these measures are all of relevance.

For parasites, some of the most important foodborne diseases are considered to be NTD by WHO, and interventions are thus included among initiatives already in place with specific goals and intentions (e.g., WHO, 2020). As an example, cysticercosis, which, as mentioned in Section 2, was estimated in 2015 to be associated with the fourth-highest DALY burden among the foodborne hazards (with only non-typhoidal *S. enterica* infections, invasive *Salmonella enterica* Typhi, and enteropathogenic *E. coli* being estimated as having higher burdens (Kirk et al., 2015; Torgerson et al., 2015)), is included in the NTD WHO roadmap (WHO, 2020) with specific intervention priorities. Among these priorities are: (1) to develop and validate specific, sensitive diagnostic tools for porcine cysticercosis, and (2) to develop a sensitive, specific point-of-care diagnostic for human taeniasis and neurocysticercosis in resource-limited settings (WHO, 2020). Two of the listed critical actions for control are the development of a high-throughput test for evaluating control programmes in resource-limited settings and mapping endemic areas, and conducting targeted interventions in areas of high endemicity. The core strategic interventions listed include: preventive chemotherapy (effective treatment of human cases of taeniasis); WASH interventions (particularly safe disposal of faeces and general hygiene in food preparation); veterinary public health measures (preventing pigs from accessing human faeces, pig vaccination, and anthelminthic treatment of pigs); and, finally, community health education on the previous interventions.

These are all measures that should limit completion of the *T. solium* life cycle, and thus reduce the burden of cysticercosis, and that should be supported. Indeed, *T. solium* is one of the few diseases that seems suitable for elimination, and the International Taskforce for Disease Eradication of the Carter Center has categorised cysticercosis as being potentially eradicable (Sadiq et al., 2024). Success in eradication of *T. solium* infection is reliant on two main points, pigs not having the opportunity to eat human faeces and humans not eating raw or undercooked pork containing the viable cysticerci larvae of pigs. However, how to measure the impact of the interventions, and ensure the continued success of regionally implemented interventions may not be so straightforward. The NTD roadmap suggests using the number of countries with intensified control in hyperendemic areas as an indicator (WHO, 2020), but although this may indicate rollout, it is hard to envisage how this could indicate the success of specific measures that have been introduced. Ring-screening (in which pigs are systematically investigated for infection by serology, and all people living within a given radius from a positive pig are treated) has been tested in endemic areas of Peru (O'Neal et al., 2014) and Zambia (Trevisan et al., 2024). Despite encouraging results, both conclude that the approach needs further refining for recommendation for use. An expert meeting report from WHO (2022) concerned with the utility of currently available diagnostic tools in public health programmes combatting *T. solium*, concluded that the diagnostic tests that are currently available are not well-suited for use in public health programmes, and that the diagnostic options for mapping and monitoring *T. solium* in public health programmes are far from optimal, largely due to sensitivity and specificity issues (WHO, 2022).

## 5 Conclusion

Determining estimates of the burden of foodborne disease associated with particular hazards provides a basis on which resource allocation can be decided and risk-management endeavours prioritised. These estimates also provide a framework in which the importance of different relevant aspects, such as education of food producers and consumers or development of sanitation and water supply infrastructure, can be highlighted.

Although the estimates published in 2015 offered a first snapshot, revising these figures a decade later provides the opportunity, not only to update the status based on new figures for occurrence (incidence or prevalence), but also to ensure that all relevant hazards have been included and also all relevant disease states that may contribute significantly to the disease burden. This may be particularly important for parasites, as not only is the diversity of those that may be transmitted by food very broad, but also the resultant diseases extend far beyond diarrhoeal illness for many of them.

Ensuring that parasites are not left behind in making these estimates is essential for ensuring that they are properly addressed in future endeavours concerned with improving food safety globally.

## CRediT authorship contribution statement

**Lucy J. Robertson:** Writing – review & editing, Writing – original draft, Visualization, Conceptualization.

## Declaration of competing interest

The authors declare the following financial interests/personal relationships which may be considered as potential competing interests:

Lucy Robertson reports a relationship with World Health Organization that includes: board membership and travel reimbursement. Lucy Robertson reports a relationship with Norwegian Scientific Committee for Food Saftety that includes: board membership. As well as being a member of the Foodborne Disease Burden Epidemiology Reference Group (FERG) of WHO - the work of which forms the backbone of this manuscript, I am also a member of the Norwegian Scientific Committee for Food Safety and the Environment, the work of which is also mentioned in this manuscript. In addition, I am Associate Editor of the Elsevier journal to which I am submitting (FAWPAR) and I am the General Secretary of the European Federation of Parasitologists which is responsible for organising the EMOP, the colloquium at which the basis of this manuscript was presented during August 2024.
